# Paradoxical facilitation alongside interhemispheric inhibition

**DOI:** 10.1007/s00221-021-06183-9

**Published:** 2021-09-02

**Authors:** Michel Belyk, Russell Banks, Anna Tendera, Robert Chen, Deryk S. Beal

**Affiliations:** 1 Department of Speech, Hearing and Phonetic Sciences, University College London, London, UK; 2 Bloorview Research Institute, Holland Bloorview Kids Rehabilitation Hospital, Toronto, ON Canada; 3 Krembil Research Institute, University Health Network, University of Toronto, Toronto, ON Canada; 4 Division of Neurology, Department of Medicine, University of Toronto, Toronto, ON Canada; 5 Department of Speech-Language Pathology, Faculty of Medicine, University of Toronto, Toronto, ON Canada

**Keywords:** Transcranial magnetic stimulation, Interhemispheric inhibition, Corpus callosum, Experimental design, Optimization, Facilitation

## Abstract

Neurophysiological experiments using transcranial magnetic stimulation (TMS) have sought to probe the function of the motor division of the corpus callosum. Primary motor cortex sends projections via the corpus callosum with a net inhibitory influence on the homologous region of the opposite hemisphere. Interhemispheric inhibition (IHI) experiments probe this inhibitory pathway. A test stimulus (TS) delivered to the motor cortex in one hemisphere elicits motor evoked potentials (MEPs) in a target muscle, while a conditioning stimulus (CS) applied to the homologous region of the opposite hemisphere modulates the effect of the TS. We predicted that large CS MEPs would be associated with increased IHI since they should be a reliable index of how effectively contralateral motor cortex was stimulated and therefore of the magnitude of interhemispheric inhibition. However, we observed a strong tendency for larger CS MEPs to be associated with reduced interhemispheric inhibition which in the extreme lead to a net effect of facilitation. This surprising effect was large, systematic, and observed in nearly all participants. We outline several hypotheses for mechanisms which may underlie this phenomenon to guide future research.

**Supplementary Information:**

The online version contains supplementary material available at 10.1007/s00221-021-06183-9.

## Introduction

A population of neurons in the primary motor cortex has axons that project across the corpus callosum to excite local inhibitory circuits in the opposite hemisphere with a net inhibitory influence on the homologous region of the brain. This pathway is the target for a developing field of brain stimulation treatments for conditions such as unilateral stroke in which the damaged hemisphere is further impaired by excessive inhibition from the healthy hemisphere (Kirton et al. 2008; Le et al. 2014; Yang et al. 2015; Eng et al. 2018). The continued development of this therapeutic approach requires a thorough understanding of the underlying neurobiology of movement control, and the mechanisms of communication between the cerebral hemispheres—namely the corpus callosum.

**Fig. 1:**
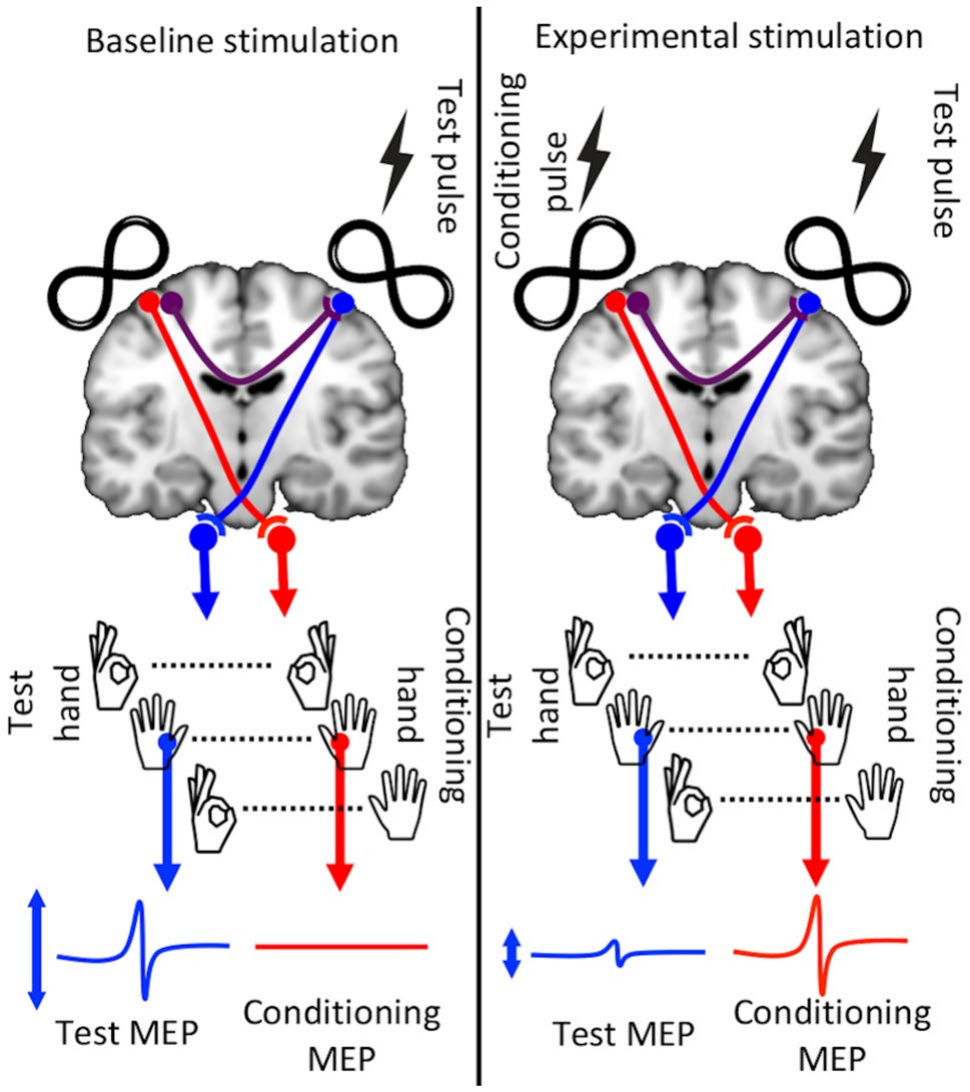
Paired-coil experiments. During *baseline stimulation* trials (left panel), test stimuli applied to the right hemisphere elicit a Motor Evoked Potential (MEP) in the left hand that is recorded as a baseline measurement. In *experimental stimulation* trials (right panel), the same test stimulus is preceded by a conditioning stimulus in the left hemisphere and elicits a smaller MEP; this reduction in MEP amplitude is taken as a measure of IHI. MEPs from the test hand (TS MEPs) and from the conditioning stimulus (CS MEPs) measure how effectively each magnetic stimulus activated the primary motor cortex

The neurophysiology of the corpus callosum can be studied using transcranial magnetic stimulation (TMS) by applying a conditioning stimulus (CS) to primary motor cortex in one hemisphere prior to applying a test stimulus (TS) in the opposite hemisphere. The conditioning stimulus reduces the magnitude of the motor evoked potentials (MEPs) from the test stimulus. This reduction in the size of the MEPs is a measure of interhemispheric inhibition (IHI; see Fig. 1).

The corpus callosum is a large band of white matter carrying axons that connect the left and right cerebral hemispheres (Witelson 1989; Hofer and Frahm 2006; Chao et al. 2009). Axons in the corpus callosum primarily project homotopically, innervating tissue near the contralateral homologue to their point of origin though a minority of transcallosal fibers also project non-homotopically (Yorke and Caviness 1975; Chovsepian et al. 2017). This pattern holds for the primary motor cortex of the precentral gyrus where the ordered representation of the muscles of the body form a somatotopic map in the gray matter of the motor cortex (Leyton and Sherrington 1917; Penfield and Boldrey 1937; Lotze et al. 2000) that is preserved in the white matter of the corpus callosum (Wahl et al. 2007; van den Heuvel and Pol 2010). Axons in the corpus callosum originate from neurons that are distinct from the giant pyramidal neurons whose axons form the corticospinal tract to carry motor commands to the peripheral nervous system (Catsman-Berrevoets et al. 1980; Leyva-Díaz and López-Bendito 2013; Chovsepian et al. 2017). The axons of the corpus callosum are excitatory, but synapse on inhibitory interneurons in the contralateral hemisphere (Kawaguchi 1992; Conti and Manzoni 1994) with a net inhibitory influence on the primary motor cortex of the contralateral hemisphere (Kuo et al. 2017; Fling et al. 2013).

Ferbert et al. (1992) reported the first experiment to probe transcallosal signaling non-invasively in humans using TMS. Ferbert et al. recorded the size of conditioned MEPs as a proportion of the size of unconditioned MEPs using TS and CS magnitudes that were above the motor threshold and with short interhemispheric intervals. Sites of stimulation that were most effective for eliciting MEPs in contralateral muscles were also the most effective sites for conditioning stimuli that inhibited MEPs for the ipsilateral muscles, consistent with homotopic communication between the motor cortices of the two hemispheres.

In addition to interhemispheric inhibition, Ferbert et al. observed periods of facilitation but noted that they were capricious, being detected in some blocks of trials but absent in others. That occurrences of facilitation were grouped in blocks rather than randomly distributed across them is suggestive of an undocumented biological mechanism. We observed a similar phenomenon while conducting a pilot experiment to optimize the design of IHI experiments (see pilot in S1). Exploratory analyses of these observations suggested that there may be an association between the size of the MEP produced by the conditioning stimuli (CS MEPs) and the degree of IHI. This observation appeared to be paradoxical; although stronger conditioning stimulus intensities increased IHI, larger CS MEPs were associated with reduced IHI contrary to expectations. We, therefore, conducted a confirmatory experiment in which MEPs were measured from both hands to determine whether this paradoxical effect of facilitation was reliable.

## Methods

### Participants

Twenty-One participants (13 female, 1 left-handed, 1 ambidextrous, ages 22–42 years) were recruited from the Bloorview Research Institute. The one left-handed participant was not an outlier in any analysis. All participants were neurologically healthy adults who gave written informed consent. Prior to the study, all participants completed a TMS safety screening form (Rossi et al. 2009) and the Edinburgh handedness questionnaire (Oldfield 1971).

### Electromyography

Adhesive bipolar electrodes (Kendall Medi-trace Foam tear-drop shape electrodes) were placed on the belly of the first dorsal interosseous (FDI) muscle and at the metacarpal-phalangeal joint of the index finger of both the left and right hands. A common ground was placed on the left ulnar styloid process. Biofeedback was continuously available to participants via an electromyogram (EMG) display. The EMG data were collected using the BrainSight™ software (v2.3.8; Rogue Research Incorporated) paired with the built-in, 2-channel EMG device. EMG signals being obtained from FDI muscles of both hands were baseline autocorrected prior to the start of each session. Live signals were amplified using the SENS-002-001 Model 2 amplifier incorporated into the BrainSight™ suite. This hardware applied a bandpass filter of 16–470 Hz. During magnetic stimulations, participants were instructed to flex the distal interphalangeal joint of the index finger against the thumb to produce pre-stimulation EMG activity of approximately 40 µV peak-to-peak.

### Selection of neural targets

The Brainsight™ neuronavigation software (v2.3.8; Rogue Research Incorporated), running on an iMac desktop computer (OS 10.13.4), was used to inform the positioning of the TMS coils. The ICBM152 MRI atlas brain (Fonov et al. 2009) was warped to fit a digital reconstruction of each participant’s head as a gross approximation of the underlying brain structure. The relative position of the participant’s head and the position of the TMS coils were continuously monitored with an integrated motion-tracking camera (Polaris Vicra, NDI).

We defined an 8-by-6 point rectangular grid of neural targets over the dorsal half of peri-central cortex in both the left and right hemisphere. Targets were spaced 5 mm apart, less deformation of the rectangular grid to conform to the contour of the brain surface. Stimulation across grids with these features are reliable localizers for the motor regions that control the muscles of the hand (Weiss et al. 2013). All stimulation targets specified a coil orientation that was perpendicular to the cortical surface and the central sulcus inducing current in an antero-medial direction (Mills et al. 1992; Balslev et al. 2007). Targets were stimulated in random order three times each, using a Magstim 70 mm (from centre to outer diameter) figure-of-eight alpha coil at 60% maximum stimulator output (MSO) with a Magstim_200_ stimulator (Magstim, United Kingdom).

A heat map of MEPs was generated over the surface of the brain with a smoothing kernel of 5 mm full-width-half-maximum and overlaid with the locations of stimulations that produced the largest MEPs. From these combined sources of information, a virtual target was placed at the optimal stimulation sites for each participant and in each hemisphere. TMS coils were then changed over to the smaller 50 mm and 40 mm Magstim alpha branding iron style figure-of-eight coils for all subsequent steps in this procedure. The optical markers which are used to track each coil were recalibrated to ensure that neuronavigation was consistent across coils. Each coil was then mounted on a passive mechanical arm, adjusted towards the position and orientation of the virtual target, and finally stimulation for each coil was triggered manually to verify that coil placements yielded MEPs in the right and left FDI muscles.

### Active motor threshold

A 50 mm Magstim alpha branding iron style figure-of-eight coil was mounted to a passive mechanical arm and navigated to the participant-individualized test neural target in the right hemisphere and a 40 mm coil was placed over the participant-individualized conditioning target in the left hemisphere. Coils were oriented perpendicular to the scalp and induced current in an anterior-medial direction as close to perpendicular to the central sulcus as possible within the constraints of space on the scalp. For some participants with smaller head sizes or more dorsal individualized targets, it was necessary to compromise either coil orientation or placement to accommodate both coils on the scalp. Choosing smaller coil sizes partially mitigated this constraint and all deviations from the ideal coil configuration were recorded by motion capture cameras. Active motor thresholds were found by identifying stimulator intensity values that produced MEPs greater than 100 µV on at least five out of ten trials (Chen et al. 2003; Rossini et al. 2015).

### Interhemispheric inhibition

The TMS coils were connected to two Magstim_200_ stimulators that were controlled by a MacMini computer (OS v10.10.5) running Python (v2.7.1) extended with the MagPy package and connected to the stimulators by custom-built cables adapted from McNair (2017). Stimulators were triggered remotely with an inter-trial interval of 10 s. Test stimuli were delivered to the right hemisphere. Conditioning stimuli were delivered to the left hemisphere prior to test stimuli. In the baseline condition, test stimuli were not conditioned. Thirty milliseconds after each unconditioned trial the conditioning coil was fired at the active motor threshold to maintain a similar rate of stimulation in both hemispheres. Conditioning stimuli were applied at 1.2 times active motor threshold, test stimuli were applied at 1.3 times active motor threshold. These parameters were selected following pilot experiments which demonstrated that they produced the largest IHI effect sizes of the parameter combinations tested (see S1). Interhemispheric intervals were tested at both 10 ms and 50 ms which correspond to short-latency IHI (SIHI) and long-latency IHI (LIHI), respectively (Ni et al. 2009).

Participants completed three blocks of stimulations each with a self-paced rest between blocks. In the separate blocks, participants were instructed to maintain (i) both FDI hand muscles pre-activated, (ii) both at rest, or (iii) pre-activated in the test hand only (see Fig. 1). Blocks were completed in counterbalanced order. The EMG signal for the 50 ms prior to stimulation was analysed to confirm that pre-contraction was of a similar magnitude within resting and within active states of the FDI and clearly distinguished between these conditions (see Table 1 and S2). Each block consisted of 60 trials (20 trials each of 10 ms SIHI, 50 ms LIHI, and unconditioned baseline stimulation) delivered in random order. To manage coil heating, pre-chilled heat sinks were applied to TMS coils between blocks (see the design in Belyk et al. 2019). One participant who had higher than usual active motor thresholds was only able to complete two of the three blocks due to coil overheating.

**Table 1:** Estimated root-mean-squared EMG (µV) with bootstrapped 95% confidence intervals (CI) for the 50 ms preceding stimulation in each condition

	Test	Conditioning
AA	8.5 (CI 7.6–9.4)	19.9 (CI 17.0–22.8)
AR	30.3 (CI 25–35.8)	5.1 (CI 4.7–5.4)
RR	2.2 (CI 2.2–2.3)	3.7 (CI 3.5–3.9)

AA: both hands active; AR: test hand active; RR: both hands at rest. The ranges of resting and active muscle pre-EMG were non-overlapping

### Analysis

We fit linear mixed models (LMMs) in R (Bates et al. 2015; Wickham 2019; R Core Team 2019) to the data to predict inhibition of peak-to-peak MEP amplitudes from fixed-effect predictors of interhemispheric interval (10 ms, 50 ms), hand configuration (both at rest, both active, test hand active), and conditioning MEP magnitude. The position, twist, and angle of both coils were included as covariates to model head movement. The model included a random slope of conditioning MEP nested within participant to assess individual variation in paradoxical facilitation. LMMs took the following form, which was selected by comparing eligible models using the Akaike Information Criterion (Harrison et al. 2018).

$$\begin{aligned} {\text{Inhibition}} & \sim {\text{interhemispheric}}\_{\text{interval }} + {\text{ hand}}\_{\text{configuration }}*{\text{ CS}}\_{\text{MEP}} \\ & + {\text{ CS}}\_{\text{position }} + {\text{ CS}}\_{\text{twist }} + {\text{ CS}}\_{\text{angle}} \\ & + {\text{ TS}}\_{\text{position }} + {\text{ TS}}\_{\text{twist }} + {\text{ TS}}\_{\text{angle}} \\ & + \, \left( {1|{\text{Participant}}} \right) \, + \, \left( {0 \, + {\text{ CS}}\_{\text{MEP}}|{\text{Participant}}} \right) \\ \end{aligned}$$

We calculated inhibition as the ratio of conditioned MEPs to the median unconditioned MEPs following common procedures. However, we observed that this approach induced highly concerning violations of standard statistical assumptions (see S3). The residuals of models of MEP ratios deviated severely from the expected distribution indicating a violation of the assumption of normality, have non-constant variance across the range of fitted values indicating a violation of the assumption of homoscedasticity, and the relation between MEPs and MEP ratios were not constant across participants further complicating their interpretation. Together these issues present serious concerns for the accuracy of p-values derived from statistical tests on MEP ratios and the validity of the inferences that are made from them. We, therefore, report a parallel analysis in which inhibition was calculated as a linear subtraction between conditioned MEPs and the median of unconditioned MEPs.

While the subtraction approach is less commonly applied in this field, it displayed few of the undesirable properties that were observed for analyses following the ratio method. The assumption of homogeneity was satisfied, although there was a minor deviation from the normality of residuals. Although LMMs are robust to minor violations of normality (Schielzeth et al. 2020), we mitigated any remaining concerns by evaluating the explanatory value of fixed-effect predictors using bootstrapped Likelihood Ratio Tests (LRTs) with 5000 simulations. Parametric bootstrapping estimates an empirical distribution from the data and makes few assumptions (Dixon 2001; Halekoh and Højsgaard 2014; Luke 2017). Fixed effect predictor variables were scaled by twice their standard deviation such that parameter estimates within the model serve as standardized measures of effect magnitudes (Gelman 2008). The data and R code for all analyses are available in the supplementary materials.

We report our results using the language of statistical clarity rather than significance (Dushoff et al. 2019), in light of the high rates of misinterpretation of the latter term. We emphasize effect sizes and confidence intervals over statistical thresholds following persistent cautions from statisticians over “bright line” thresholds (Wasserstein and Lazar 2016; Wasserstein et al. 2019).

## Results

The mean active motor thresholds were 52%MSO (11.2 SD) for the test hemisphere and 53%MSO (11.5 SD) for the conditioning hemisphere. Mean unconditioned test MEPs were 219 (217 SD) μV, 455 (337 SD) μV, and 410 (320 SD) μV, respectively, with both hands at rest, only the test hand active, or both hands active, respectively.

### Analysis of differences

A greater degree of inhibition was observed for LIHI at 50 ms compared to SIHI at 10 ms (LRT = 36.2, *p* = 0.0002, standardized estimate = 31.5; positive values indicate greater inhibition). The model selected for this analysis did not assess whether interhemispheric interval interacted with the configuration of the hands. The configuration of the hands affected interhemispheric inhibition (LRT = 39.3, *p* = 0.0002, see Table 2) with reduced inhibition when the test hand was pre-activated (standardized estimate = − 2.6) and increased inhibition when both hands were pre-activated (standardized estimate = 43.4). However, in light of significant interactions with other predictors (see below) caution is advised when interpreting these main effects in light of the presence of interactions.

**Table 2:** Model estimates and 95% confidence intervals (CI) of inhibition observed in each condition

Condition	IHI (ms)	Inhibition (μV)
AA	10	45.3 (CI 25.1–65.6)
AA	50	76.8 (CI 56.3–97.6)
AR	10	− 16.8 (CI − 33.9– − 0.07)
AR	50	14.7 (CI − 2.45–31.7)
RR	10	21.9 (CI 5.35–38.3)
RR	50	53.4 (CI 36.6–69.6)

AA: both hands active; AR: test hand active; RR: both hands at rest at two interhemispheric intervals (IHI). Positive values indicate increased interhemispheric inhibition. These estimates are taken assuming the lowest levels of CS MEPS (i.e., in the absence of facilitation). Interhemispheric inhibition was strongest for 50 ms IHI and when both hands were pre-activated. Note that these values interact strongly with CS MEPs and should be interpreted with caution

**Fig. 2:**
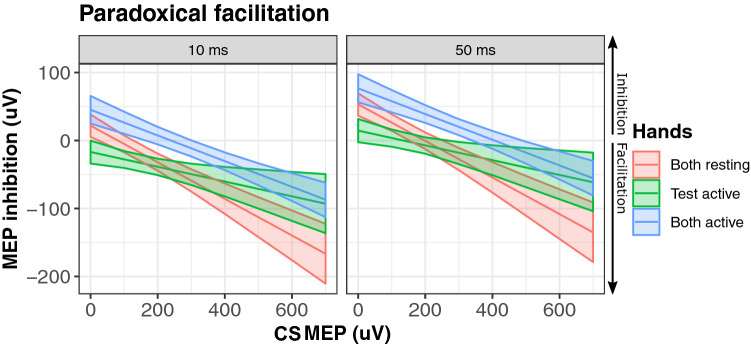
Paradoxical facilitation from MEP differences. MEP amplitudes from the conditioning stimulus (CS MEPs) strongly predicted reduced inhibition of MEPs produced by test stimulus (TS MEPs). Larger values indicate inhibition. This effect is apparent across configurations of muscle pre-activation. When CS MEPs were sufficiently large, the effects of interhemispheric inhibition were reversed to a net facilitation. Blocks with both hands at rest (red), only the test hand active (green), or both hands active (blue) are plotted separately. Shaded areas represent 95% prediction intervals

**Fig. 3:**
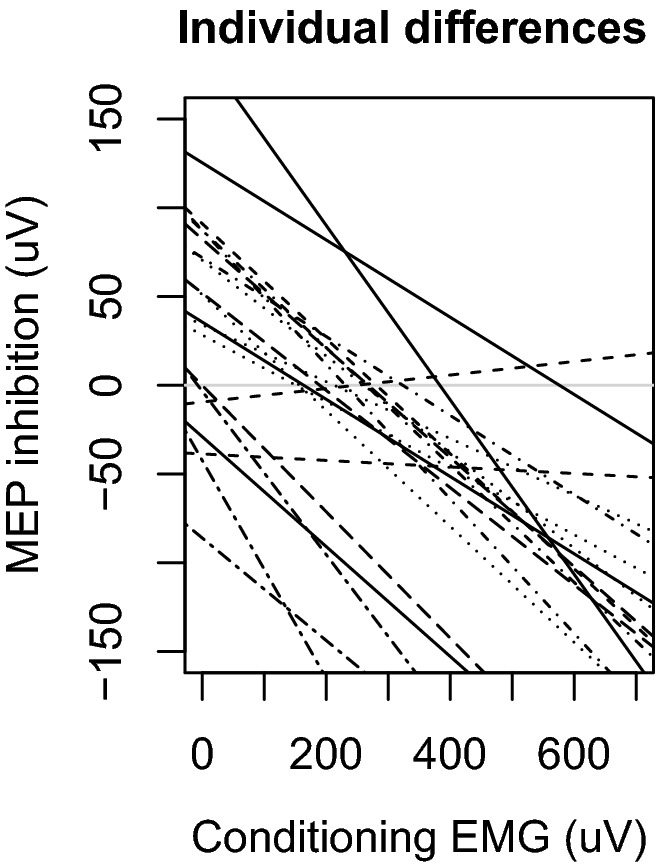
Individual differences in paradoxical facilitation from MEP differences. The association between contralateral inhibition and CS MEP amplitudes while both hands were at rest are plotted separately for each participant. Participants with negative slopes demonstrated paradoxical facilitation. While participants varied somewhat in the degree to which paradoxical facilitation was observed, this variation was small relative to the size of the facilitatory effect and facilitation was observed in 19 of 21 individuals. MEP inhibition values below 0 indicate a net effect of facilitation

We observed that CS MEPs had a strong influence on inhibition (LRT = 7.5, *p* = 0.009, standardized estimate = − 108.7). From the magnitude of the standardized estimates it is clear that this effect was considerably stronger than the effects of either interhemispheric interval or hand configuration. We refer to this effect as paradoxical facilitation because the CS did elicit inhibition, and yet the CS also produces facilitation in proportion to its effectiveness in driving its own target muscle (see Fig. 2; see S3 for visualizations of raw data). Paradoxical facilitation interacted strongly with the configuration of the hands (LRT = 10.9, *p* = 0.005). Paradoxical facilitation was observed less strongly when both hands where pre-activated (standardized estimate = 32.8), and when only the test hand was pre-activated (standardized estimate = 64.8) relative to both hands at rest. An examination of the random slopes of conditioning MEP with participants demonstrates that individual differences were comparatively small and paradoxical facilitation was observed in 19 of 21 participants (effects ranging from − 251.7 to 29.4, median of − 113.5; see Fig. 3). Qualitatively similar findings were observed from parallel analyses using robust correlations in lieu of linear mixed models (see S4) and including pre-stimulation EMG as covariates (see S2) demonstrating that these findings are robust to researcher degrees of freedom.

### Analysis of ratios

A parallel analysis of ratio transformed data yielded qualitatively similar results, though with reduced statistical power and severe violations of common statistical assumptions (see S5). We observed a greater degree of inhibition for LIHI at 50 ms than SIHI at 10 ms (LRT = 11.7, *p* = 0.0006, standardized estimate = − 0.21; negative values indicate greater inhibition). The configuration of the hands did not have a clear effect on inhibition ratios (LRT = 3.9, *p* = 0.14) with neither both hands pre-activated (standardized estimate = − 0.15) nor only the test hand pre-activated (standardized estimate = − 0.02) having notable effects on inhibition ratios relative to both hands at rest.

**Fig. 4:**
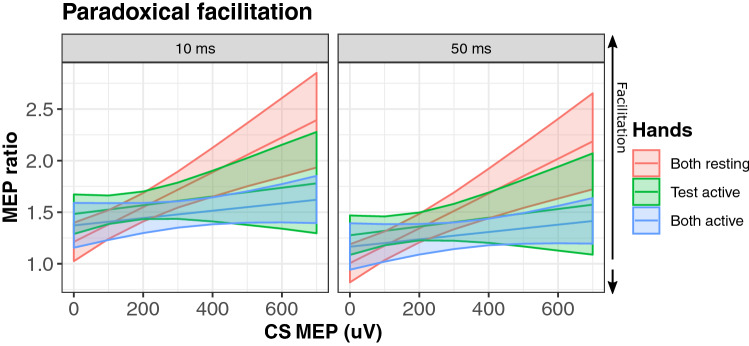
Paradoxical facilitation from MEP ratios. MEP amplitudes from the conditioning stimuli (CS MEPs) strongly predicted reduced inhibition of MEPs produced by test pulses (TS MEPs). Larger values indicate facilitation. Blocks with both hands at rest (red), the test hand active (green), or both hands active (blue) are plotted separately. Shaded areas represent 95% prediction intervals

**Fig. 5:**
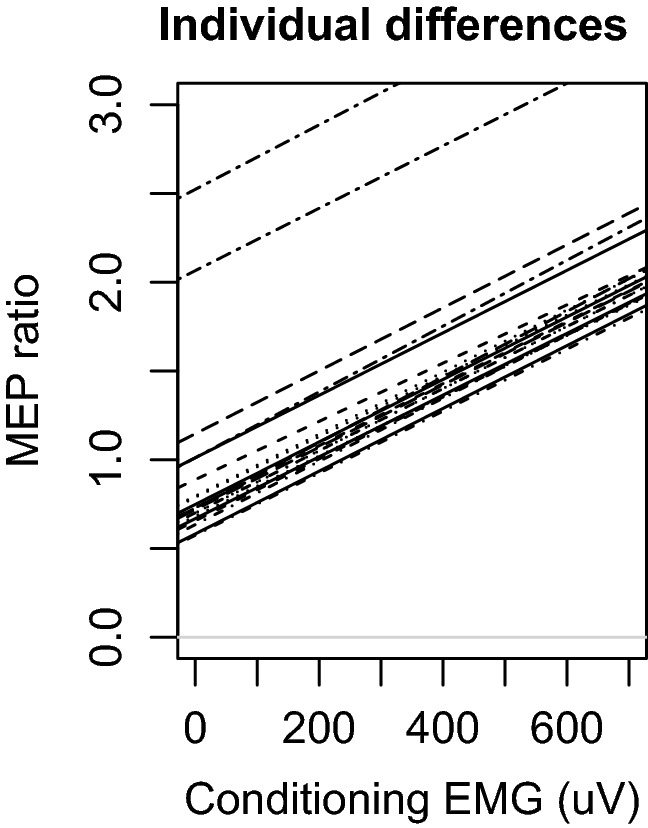
Individual differences in paradoxical facilitation from MEP ratios. The association between contralateral inhibition and CS MEP amplitudes while both hands were at rest are plotted separately for each participant. Participants with positive slopes demonstrated paradoxical facilitation. Using this metric there was only minimal variation in the degree to which participants demonstrated paradoxical facilitation, and a clear facilatory effect was observed in all participants. MEP ratio values greater than 1 indicate a net effect of facilitation

Paradoxical facilitation remained the strongest detectable effect in the analysis of ratios (LRT = 8.6, *p* = 0.006, standardized estimate = 0.73). Paradoxical facilitation interacted with the configuration of the hands (LRT = 8.4, *p* = 0.02). Paradoxical facilitation was observed less strongly when both hands where pre-activated (standardized estimate = − 0.56), and when only the test hand was pre-activated (standardized estimate = − 0.52) relative to both hands at rest (see Fig. 4). An examination of the random slopes of conditioning MEP with participant demonstrates that individual differences where comparatively small and paradoxical facilitation was observed in all 21 participants (effects ranging from 0.69 to 0.76, median of 0.73; see Fig. 5).

### Distributions of MEPs

**Fig. 6:**
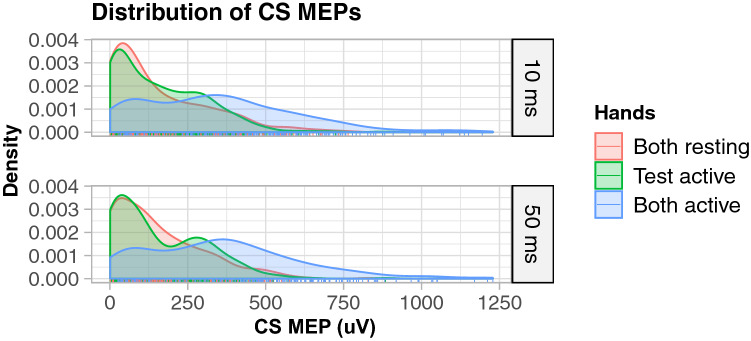
Distribution of CS MEP amplitudes. Conditioning stimuli MEPs (CS MEPs) were strongly left skewed such that large CS MEPs, which have a large facilitatory influence, were relatively infrequent. The comparative rarity of large CS MEPs may explain why a net effect of interhemispheric inhibition can still be observed by taking the mean across observations. Density (*y*-axis) is a measure of the abundance of observation across values of the *x*-axis for the purpose of visualising the shapes of distributions

In light of the strength of facilitation associated with the CS MEPS, it may be surprising that a net effect of inhibition can be observed on average. An examination of the distribution of CS MEPs reveals a strong leftward skew towards mild activation of the FDI muscle (see Fig. 6). Instances of CS MEPs of a sufficiently large size to reverse the inhibitory effect of contralateral stimulation are rare, and, therefore, for the majority of trials the balance lands in favor of inhibition.

## Discussion

We predicted that larger CS MEPs would be associated with increased IHI as an index of how effectively contralateral motor cortex was stimulated since greater conditioning stimulus magnitudes are associated greater interhemispheric inhibition (Liang et al. 2014; Wahl et al. 2016) and with larger CS MEPs. However, during a pilot experiment in which we tested a range of parameters to optimize a common experimental design that probes IHI, we observed a strong tendency towards the opposite. Larger CS MEPs are associated with reduced interhemispheric inhibition which in the extreme leads to a net effect of facilitation. The present study demonstrated that this paradoxical facilitation (1) is reliable since it was observed in all or nearly all participants, (2) has a greater influence on observations than do commonly manipulated experimental parameters such as pre-activation of the target muscles, and (3) can be modulated by the pre-activation of muscles on one or both sides of the body.

The present study used a relatively low CS intensity of 120% active motor threshold to mitigate coil heating. Further studies are needed to investigate whether the same relationship between CS MEP amplitude and IHI can be observed with higher CS intensities, which are expected to evoke stronger IHI. Our pilot experiments (see S1) indicated that while experimental parameters should be chosen based on the neurophysiological mechanisms under study (Irlbacher et al. 2007; Udupa et al. 2010), inhibition increased for both SIHI and LIHI up to the strongest CS that were pragmatic for experimental design, consistent with previous studies (Harris-Love et al. 2007; Ni et al. 2009). Hence our findings are consistent with previous observations that stronger CS magnitudes increase interhemispheric inhibition (Liang et al. 2014; Wahl et al. 2016). Stronger test stimuli yield larger TS MEPs, which may permit a larger maximum reduction in TS MEPs, though greater stimulation magnitudes may strain participant tolerability and coil heat dissipation (Belyk et al. 2019). We observed the strongest interhemispheric inhibition effects on trials where strong conditioning stimuli elicited relatively small CS MEPs, and the strongest facilitation when weak conditioning stimuli elicited relatively strong CS MEPs.

It is a standard practice among IHI studies to analyse inhibition as the ratio of conditioned and unconditioned MEP amplitudes following the approach of Ferbert et al. (1992). We observed qualitatively similar results with and without a ratio transform, although we note that the ratio form fails to meet standard statistical assumptions (see S3) and, as such, we do not recommend that approach (Stark and Saltelli 2018).

### Paradoxical facilitation

The earliest TMS study of interhemispheric inhibition observed occasional instances of facilitation rather than inhibition (Ferbert et al. 1992). Ferbert et al. observed periods of facilitation, which they associated with very short latency interhemispheric intervals, but noted that it was capricious, being present unreliably in some blocks of trials. Another study also observed interhemispheric facilitation, but only for ISIs shorter than were tested in the present experiment (Hanajima et al. 2001). Ni et al. (2009) observed that participants who had larger CS MEPs exhibiting greater inhibition. However, those findings reflect differences between participants and not the within participant variation on a trial-by-trial basis reported in the present study. To our knowledge, the relationship between trial-by-trial variation in CS MEP amplitudes and IHI at a constant CS intensity has not been reported.

It is likely that both inhibitory and facilitatory circuits are engaged in paired-coil TMS studies and that the observed MEPs are impacted by both processes. For instance, we observed that stronger conditioning stimuli did increase inhibition, however, they also produced larger CS MEPs, which we have demonstrated are associated with facilitation. Hence, competing mechanisms may result in net inhibition or facilitation depending on the balance of contributing factors (Chen 2004).

We suggest that the source of this facilitation may be found in the central nervous system (CNS) rather than the peripheral nervous system. Pre-contraction increased the frequency of high amplitude CS MEPs while reducing the slope of the facilitation effect. Previous studies have found that pre-activation of one hand increases IHI bidirectionally (Nelson et al. 2009). This constellation of findings may indicate that large CS MEPs are a result of a process that leads to facilitation but not its immediate cause.

Muscle pre-contractions can impact cortical processes. For instance, SIHI and LIHI are modulated to a different extent by pre-contraction of the conditioning hand (Uehara et al. 2014). We further observed that paradoxical facilitation is modulated by pre-contraction of either the conditioning or test hand. However, we also observed reduced magnitudes of inhibition for SIHI relative to LIHI, after controlling for the pre-activation of the hand muscles and for the magnitude of CS MEPS. This finding may indicate that mechanisms driving differences between SIHI and LIHI are distinct from the mechanisms driving paradoxical facilitation. For instance, LIHI is distinct from SIHI in being mediated by GABAb receptors (Irlbacher et al. 2007) which argues against the role of such a mechanism in mediating paradoxical facilitation. We outline three hypotheses for CNS mechanisms that may mediate paradoxical facilitation.

### Spatial anticorrelation: corticospinal and transcallosal neuronal populations with different susceptibilities to stimulation

A defining feature of the primary motor cortex is the presence of giant pyramidal neurons in cortical layer V with axons that form the corticospinal tract (Cambell 1904; Brodmann 1909). A separate population of neuronal cells spanning cortical layers II, III and V form the corpus callosum pathway that mediates interhemispheric inhibition (Catsman-Berrevoets et al. 1980).

The pattern of electrical activity induced in the cortex by magnetic stimulation varies non-linearly over cortical space (Deng et al. 2013; Laakso et al. 2014) and is strongly influenced by the position and orientation of the underlying tissue (Wagner et al. 2004) which is reflected in the resulting MEPs (Mills et al. 1992; Balslev et al. 2007).

It has previously been hypothesized that the distribution of trans-callosal and corticospinal circuits may be only partially overlapping (Avanzino et al. 2007). To the extent that neurons that form the corticospinal tract differ on average in either position or orientation from neurons that form the corpus callosum, we hypothesize that individual trials may be better optimized to drive one or the other of these pathways, hence inducing the unexpected negative correlation between CS MEPS as a measure of how effectively the corticospinal neurons were driven and IHI as a measure of how effectively the corpus callosum was stimulated.

The different distributions of cell bodies that originate these two pathways may lead stimuli that are effective at stimulating the corticospinal tract to be less effective at stimulating the corpus callosum. If this is the case, then paradoxical facilitation does not reflect the functioning of interhemispheric fibers passing through the corpus callosum, though it strongly impacts experiments that are commonly used to study this pathway. While the present study statistically controlled for deviations in the position and orientation of stimulator coils using linear models, tests of this hypothesis will require complimentary evidence from electrical field mapping.

### Temporal anticorrelation: phases of neuronal oscillation

Alternatively, we hypothesize that the paradoxical facilitation effect may be the result of fluctuations in brain states. Oscillatory rhythms are an organizing principle of brain dynamics (Buzsáki and Draguhn 2004; Fries 2005). For example, the mu-rhythm is a salient oscillation in the range of 8–13 Hz. TMS applied to motor cortex elicits larger MEPs at the negative peak of mu-oscillations than at the positive peak, demonstrating that endogenous fluctuations in brain states may influence corticospinal excitability (Zrenner et al. 2018; Schaworonkow et al. 2019) although see Madsen et al. (2019). Whether mu-oscillations affect interhemispheric inhibition remains to be studied and it is not known whether cycles of oscillation are synchronized between the left and right motor cortex. Regardless, the time between conditioning and test stimuli may lead to complex interactions as magnetic stimulations occur at different oscillatory phases in either hemisphere.

### Cross-facilitation: centre-surround excitation and inhibition

Carson (2020) proposed that excitatory transcallosal fibers serve an integrative function that co-opts the processing capacities of the two hemispheres. This model proposes that callosal fibers synapse directly onto pyramidal neurons as well as onto inter-neurons that inhibit surrounding pyramidal neurons. This center-surround topology is reminiscent of well-documented circuits for sensory processing which have the desirable attribute of sharpening neuronal receptive fields. Such a mechanism which combines excitatory and inhibitory communication between the hemispheres may be consistent with the paradoxical facilitation observed in the present study.

## Conclusion

The application of a conditioning stimulus to the contralateral hemisphere leads to interhemispheric inhibition, however, larger CS MEPs are strongly associated with interhemispheric facilitation. When CS MEPs are sufficiently large, this effect can overpower interhemispheric inhibition, leading to a net effect of facilitation. This paradoxical facilitation has a larger influence over measured TS MEPs than commonly studies factors such as conditioning stimulus magnitude, and is highly reliable across participants.

## Supplementary Information

Below is the link to the electronic supplementary material.

Supplementary file1 (DOCX 1837 kb)

Supplementary file2 (DOCX 76 kb)

Supplementary file3 (DOCX 184 kb)

Supplementary file4 (DOCX 27 kb)

Supplementary file5 (DOC 869 kb)

Supplementary file6 (CSV 317 kb)

Supplementary file7 (R 14 kb)

Supplementary file8 (CSV 774 kb)

Supplementary file9 (R 23 kb)

Supplementary file10 (PY 11 kb)

Supplementary file11 (R 15 kb)

Supplementary file12 (R 7 kb)

Supplementary file12 (RDATA 2595 kb)

## Data Availability

All data are available in supplementary materials.

## Code availability

All data collection and analysis code are available in supplementary materials.
