# Supplementary material for: Paradoxical facilitation alongside interhemispheric inhibition
Source: Exp Brain Res. 2021 Sep 2;239(11):3303–13. doi: 10.1007/s00221-021-06183-9 (PMC8541949; doi:10.1007/s00221-021-06183-9)
Supplement: Supplementary file 1 — Supplementary file1 (DOCX 1837 kb) [file 221_2021_6183_MOESM1_ESM.docx]

This document describes a pilot experiment whose aim was to derive optimal experimental parameter combinations for IHI experiments. Exploratory analyses of these data motivated the study in the main body of text.

**Methods (See main text for remaining methods)**

The TMS coils were connected to two Magstim_200_ stimulators that were controlled by a MacMini computer (OS v10.10.5) running Python (v2.7.1) extended with the MagPy package and connected to the stimulators by custom built cables adapted from McNair (2017). Stimulators were triggered remotely with an inter-trial interval of 10 s. Test pulses were delivered to the right hemisphere. Conditioning pulses were delivered to the left hemisphere 10 ms or 50 ms prior to test pulses. In the baseline condition, test pulses were not conditioned. Thirty milliseconds after each unconditioned trial the conditioning coil was fired at motor threshold to maintain a similar rate of stimulation in both hemispheres. Conditioning pulses were delivered at 0.9, 1, 1.1, or 1.2 times motor threshold. Test pulses were delivered at 1, 1.1, 1.2, or 1.3 times motor threshold. The combination of IHI (two levels), test-pulse magnitude (four levels), and conditioning-pulse magnitude (four levels) resulted in 32 experimental conditions, plus four test-only baseline conditions (one at each test-pulse magnitude). Each condition was repeated five times for a total of 180 trials in random order. Participants rested for a self-determined duration after 60 and 120 trials.

**Fig. S1.1** Paired-coil experiments. During *baseline stimulation* trials, test pulses applied to the right hemisphere elicit a Motor Evoked Potential (MEP) in the left hand that is recorded as a baseline measurement. In *experimental stimulation* trials, the same test pulse is preceded by a conditioning pulse in the left hemisphere and elicits a smaller MEP; this reduction in MEP amplitude is taken as a measure of IHI. MEPs measured from the test hand (tMEPs) and from the conditioning hand (cMEPs) measure how effectively each pulse activated primary motor cortex

*Analysis*

We calculated IHI as the reduction in the amplitude of test-pulse MEPs (tMEPs) for conditioned relative to unconditioned pulses [tMEP_conditioned_ - tMEP_unconditioned_]. The unconditioned tMEP was estimated separately for each participant and each test pulse power, as the median tMEP in the absence of a conditioning pulse. Previous studies have calculated IHI as the ratio of conditioned and unconditioned MEPs, and a parallel analysis using this approach is therefore provided for comparison (see supplementary materials). Deviations from the ideal coil position, rotation, and angle of approach were centered and scaled separately for each coil and each participant. Each distribution of coil deviations was visually inspected to identify outliers for removal. Four to 36 trials per participant were removed due to either an excessive of movement or motion tracking failure.

We fit the data using a linear mixed-effects model in R (Bates et al. 2015; Wickham 2017; R Core Team 2019) with fixed effects of conditioning pulse power (0.9, 1.0, 1.1. or 1.2 x MT), test pulse power (1.0, 1.1., 1.2 or 1.3 x MT), ISI (10 or 50 ms), and the size of the MEP produced by the conditioning pulse (cMEPs). Model selection was performed by iteratively fitting models and dropping terms to determine which subset of models could be successfully fit. Selection from among fitable models was informed by Akaike Information Criterion (AIC) with the constraint that fixed effect predictors of primary theoretical interest must be retained. The explanatory value of fixed effect predictors were evaluated using bootstrapped Likelihood Ratio Tests (LRT) with 5000 simulations (Dixon 2001; Halekoh and Højsgaard 2014; Luke 2017). The data and R code for this analysis are available in the supplementary materials.

We report our results using the language of statistical clarity rather than significance (Dushoff et al. 2019), in light of the high rates of misinterpretation of the latter term. We emphasize effect sizes and confidence intervals over statistical thresholds in light of persistent cautions from statisticians over “bright line” thresholds (Wasserstein and Lazar 2016; Wasserstein et al. 2019).

**Results**

The median motor thresholds were 46% maximum stimulator output (MSO; Range = 40-58) in the test hemisphere and 57% MSO (range= 40-70) in the conditioning hemisphere. Median MEPs in the test only baseline increased with test pulse power from 1.1xMT: median = 75 μV, range = 9-70, 1.2xMT: 141 μV range = 6-376, 1.3xMT: 333 μV range = 53-650, 1.4xMT: 436 μV range = 33-1061).

*Effects of stimulation parameters on IHI*

On average, MEPs in the test hand were inhibited by 48.2 (CI = [21.0, 58.9]) μV. Inhibition was greater with increasing conditioning pulse power (LRT = 86.5, p = 0.001, estimate = 37.2, CI = [26.4, 47.5]), test pulse power (LRT = 57.2, p = 0.001, estimate = 27.9, CI = [20.7, 34.4]), and IHI of 50 ms compared to 10 ms (LRT = 51.8, p = 0.001, estimate = 37.6, CI = [19.0, 56.1])]. The effect of test pulse power may have increased with increasing conditioning pulse power though the evidence is unclear (LRT = 3.8, p = 0.054, estimate = 7.1, CI = [-0.1, 14.0]). These effects are summarized in Figure 2. Further interactions between these factors were omitted from the model as informed by AIC scores to facilitate model fitting.

**Fig. S1.2** Heat map of experimental parameters. Coloured cells indicate the degree of inhibition observed for each combination of test pulse power (x-axes), conditioning pulse power (y-axes), and interstimulus interval (IHI; panels). There was a clear pattern of stronger test pulses and stronger conditioning pulses to yield a greater degree of inhibition. This pattern holds across intervals, though inhibition was more pronounced at interstimulus intervals of 50 ms than 10 ms.

*Paradoxical facilitation*

We hypothesized that inhibition would be strongest for trials in which the conditioning pulse strongly stimulated the underlying motor cortex, as measured by MEPs resulting from the conditioning pulse (cMEPs). However, we observed strong evidence for the opposite relation. Though stronger conditioning pulses did elicit more inhibition, larger cMEPs were associated with reduced inhibition (LRT = 23.9, p = 0.002, estimate = -0.39, CI = [-0.47, -0.30]). Each 1 μV increase in cMEP on average produced a 0.39μV decrease in IHI. Since, cMEPs were sometimes large, the conditioning pulse had a net facilitatory effect in some cases (see Figure 3). The effect of cMEP was slightly mitigated with increasing conditioning pulse power (LRT = 11.6, p = 0.002, estimate = 0.083, CI = [0.035, 0.13]) and with longer IHI (LRT = 6.9, p = 0.004, estimate = 0.087, CI = [0.021, 0.15]). Qualitatively similar results were achieved when data were treated as ratios rather than differences, though the ratio approach was prone to severe violations of the assumptions of statistical tests (see supplementary materials).

**Fig. 3** Paradoxical facilitation. MEPs in the right hand that resulted from the conditioning pulse applied to the left hemisphere (cMEPs) strongly predicted reduced inhibition of MEPs produced by test pulses (tMEPs). This trend holds across values of test pulse power (TPower), conditioning pulse power (CPower), and interhemispheric interval (IHI). Higher conditioning pulse powers increased inhibition (as demonstrated by differences in intercepts) while larger cMEPS reduced inhibition (as demonstrated by negative slopes). Y-axis values below zero indicate a net effect of facilitation. Shaded areas represent 95% confidence intervals.

The linear mixed models included a random slope of cMEP, permitting the effect of cMEP to be estimated separately for each participant. Every participant in the dataset had a negative slope, indicating that the effect of facilitation was robust across the dataset. Figure 4 plots the slope of the relationship between IHI and cMEPs separately for each participant and demonstrates that this surprising association was observed in every individual in the sample.

**Fig. 4** Individual differences. The association between IHI and cMEPs are plotted separately for each participant. While participants varied slightly in the degree to which paradoxical facilitation was observed, facilitation was observed in every individual. Values below 0 indicate a net effect of facilitation

CMEPs were strongly skewed towards the lower tail such that small cMEP magnitudes were most common (see Figure 5). Hence, large cMEPs that produced sufficient facilitation to counteract IHI were uncommon and a net inhibitory effect of the conditioning pulse was still observed.

**Fig. 5** Distribution of cMEPs Conditioning pulse MEPs (cMEPs) were strongly left skewed such that large cMEPs whose facilitatory influence may overpower the effect of inhibition were relatively infrequent. However, they increase in frequency with increasing conditioning pulse power (CPower).
