# Supplementary material for: Paradoxical facilitation alongside interhemispheric inhibition
Source: Exp Brain Res. 2021 Sep 2;239(11):3303–13. doi: 10.1007/s00221-021-06183-9 (PMC8541949; doi:10.1007/s00221-021-06183-9)
Supplement: Supplementary file 2 — Supplementary file2 (DOCX 76 kb) [file 221_2021_6183_MOESM2_ESM.docx]

We quantified pre-stimulation EMG by DC subtracting and taking the area under the curve for the 50 ms preceding stimulation (Figure A). Pre-stimulus EMG was not available for 2 participants for whom those measurements were not saved to disc. The following analyses are therefore based on the 19 participants for whom pre-stimulation EMG recordings were available.

1. **Verifying pre-contraction**

In all cases, pre-stimulus EMG followed a gamma distribution which is the expected distribution for measurements with a theoretical range from <0 to infinity. It is clear from visual inspection that hands which were at rest have values clustered near the minimum of this scale, while hands that were active had a strong positive skew.

Figure A: Density plots of pre-stimulation EMG prior to CS MEPS (Condition) and TS MEPS (Test), with both hands active (AA), the test-hand active (AR), both hands at rest (RR).

Differences and similarities between Gamma distributions can be quantified by comparing the coefficients of generalised linear mixed models. We constructed a model of the following form, which has fixed effects following the structure of Figure A in this document as well as the maximal compliment of Random slopes within participant. Explicit tests demonstrate broad consistency within the Active and Resting states and large differences between them, as expected.

PreEMG ~ Coil*Condition + (1+Coil*Condition | Participant),

family=Gamma(link="log")

Within the AA condition, pre-stimulation EMG for the Test coil was not significantly different than the Conditioning coil (estimate -0.145, t = -1.13, p = 0.25)

Within the RR condition, pre-stimulation EMG for the Test coil was not significantly different than the Conditioning coil (estimate -0.215, t = -1.01, p = 0.31)

Within the AR condition, pre-stimulation EMG for the Test coil was significantly greater than the Conditioning coil (estimate 1.19, t = 4.14, p < 0. 001). This corresponds to an expected differences of exp(1.19) = 3.28 uV between the Active and the Resting hand.

A comparison of the conditioning hand when at rest in (AR) and the same hand at rest in (RR) revealed no difference (estimate = -0.08, t = -1.28, p = 0.20)

A comparison of the test hand when active in (AR) and the same hand active in (AA) revealed a significant differences, although we note that the size of this differences was small, having a magnitude similar to the non-significant rests reported above (estimate = -0.23, t = -2.12, p = 0.034). As in the main text we caution against rigid “bright line” interpretation based on p-values.

Figure B: Estimates and 95% confidence intervals for pre-stimulation EMG, demonstrating a clear overlap of estimates within Active hands, and within Resting hands, as well as a clear separation between Active and Resting hands.

1. **Testing implications for paradoxical facilitation**

We refit the model of MEP differences reported in the main text, with the addition of continuous covariates for the pre-stimulation EMG for both the Test Hand and the Conditioning hand coil.

IHI LRT = 30.9, p <0.001 (estimate = 31.8, CI = 20.7­­ – 42.8)

Condition LRT = 14.3, p = 0.002 (AR: estimate = -21.9, CI= -39.8 ­– -3.99)

(RR: estimate = -34.0, CI= -52.8 ­– -15.24)

*CS MEP LRT = 11.3, p = 0.002 (estimate -109.6, CI = -152.2 – -65.9)*

Importantly, the model estimate for the paradoxical facilitation effect (CS MEP) was of comparable size to reports in the main text.

The degree of precontraction for the Test hand reduced inhibition

preEMG-test (estimate = -27.8, CI = -40.1 – -15.6)

preEMG-conditioning (estimate = 5.4, CI = -8.9 – 19.8)

Due to the high degree of covariance between Condition (AA, AR, RR) and pre-stimulation EMG, permutation tests for the Condition:CS MEP interaction experience high rates of singular fits. Tests of this interaction are therefore not feasible with this model.

Due to i) the reduced facility for testing interactions of theoretical interest, ii) the reduced sample size, and iii) similarity with the findings of the simpler model, we retain our original model without the addition of pre-stimulation EMG predictors in the main text.

Equivalent refitting was not performed for the ratio model, as we have serious misgivings about modelling ratio data and see little utility in promoting their continued use in this field.
