# Supplementary material for: Paradoxical facilitation alongside interhemispheric inhibition
Source: Exp Brain Res. 2021 Sep 2;239(11):3303–13. doi: 10.1007/s00221-021-06183-9 (PMC8541949; doi:10.1007/s00221-021-06183-9)
Supplement: Supplementary file 3 — Supplementary file3 (DOCX 184 kb) [file 221_2021_6183_MOESM3_ESM.docx]

Figure S2.1: Individuals data points and regression lines for interhemispheric intervals of 10 ms. Each panel presents data from a single participant for each of the three configurations of hand pre-activation.

Figure S2.2: Individuals data points and regression lines for interhemispheric intervals of 50 ms. Each panel presents data from a single participant for each of the three configurations of hand pre-activation.
