# Supplementary material for: Paradoxical facilitation alongside interhemispheric inhibition
Source: Exp Brain Res. 2021 Sep 2;239(11):3303–13. doi: 10.1007/s00221-021-06183-9 (PMC8541949; doi:10.1007/s00221-021-06183-9)
Supplement: Supplementary file 4 — Supplementary file4 (DOCX 27 kb) [file 221_2021_6183_MOESM4_ESM.docx]

**Wilcox’s Percentage Bend**

We present an alternative analysis using robust correlation with Wilcox’s Percentage Bend as an alternative to the more complex linear mixed models (LMM) method presented in the main text. This approach makes fewer assumptions about the data at the expense of statistical power and the capacity to explicitly control for extraneous sources of variation such as drift in coil positions.

Correlations between conditioning stimulus MEPs and inhibition of test stimulus MEPs were computed separately for each interhemispheric interval (10ms, 50ms), each configuration of the hand (both resting, test only active, both active), and each participant (n=21). In this analysis positive r-values would indicate that larger conditioning stimulus MEPs increased interhemispheric inhibition, negative r-values indicate that larger conditioning stimulus MEPs decreased interhemispheric inhibition (i.e., facilitation). Computations were performed using R (R Core Team, 2020) supplemented by functions from the asbio package (Aho, 2020).

This analysis yielded qualitatively similar results to the LMMs presented in the main text of this article. Correlations were negative across interhemispheric intervals and hand configurations, indicative of facilitation. Consistent with findings from the LMMs, the facilitation effect was weakest when only the test hand was active.

Figure S3.1: The mean percentage bend r-values across participants with 95% confidence intervals.

**References**

Ken Aho (2020). asbio: A Collection of Statistical Tools for Biologists. R package version 1.6-7. https://CRAN.R-project.org/package=asbio

R Core Team (2019). R: A language and environment for statistical computing. R Foundation for Statistical Computing, Vienna, Austria. https://www.R-project.org/.

Wilcox, R. R. (1994). The percentage bend correlation coefficient. *Psychometrika*, *59*(4), 601-616.
