# Supplementary material for: Paradoxical facilitation alongside interhemispheric inhibition
Source: Exp Brain Res. 2021 Sep 2;239(11):3303–13. doi: 10.1007/s00221-021-06183-9 (PMC8541949; doi:10.1007/s00221-021-06183-9)
Supplement: Supplementary file 5 — Supplementary file5 (DOC 869 kb) [file 221_2021_6183_MOESM5_ESM.doc]

**Assumption checking ratio transforms versus inhibition by subration**

Parallel analyses are reported in the main body of the text are based on a calculation of interhemispheric inhibition by method of subtraction (inhibition = MEP_unconditioned_ - MEP_conditioned_) and by the method of ratios (inhibition_ratio = MEP_conditioned_ / MEP_unconditioned_).

After transformation to ratios, IHI measurements have several undesirable statistical properties which interpreting these results problematic.

*We note that the ratio method is presented for historical comparison only. The analysis of ratios severely violates standard statistical assumptions and inferences made from such analyses are liable to be invalid.* While many researchers in this field believe that ratios may be more “clinically meaningful”, *the scientific health of this field will benefit from discontinuing this practice in favour analyses that are inferentially meaningful.*

1. The distribution of residuals is skewed. This can make measures of central tendency, such as the mean, misleading.

Fig. 4.1: Density plots of model residuals from the ratio method (left) and subtraction method (right).

1. This induces violations of the assumption of normality rendering p-values derived from tests that make this assumption inaccurate. Note the particularly strong deviations in the upper tails. The difference method provides residuals that are much improved but still imperfect.

Ratios Differences

Fig. 4.2: Quantile-quantile plots for residuals of the ratio method (left) demonstrating severe violations of normality and the subtraction method (right) demonstrating modsest violations of normality.

1. These plots show the residuals (y-axis) across the range of values fitted by the model. Linear models assume that the observations ware stationary (i.e., have similar means across values of the x-axis), which is approximately satisfied in both cases. They also assume that the variance of residuals are approximately constant across fitted values, which is severely violated by the ratio form. These diagnostics indicate a strong violation of the assumption of homoscedasticity (sometimes called homogeneity of variance) for the ratio model rendering p-values from that model invalid.

Fig. 4.3: Dispersion plots demonstrating that following the ratio method (left) dispersion increases with fitted value (i.e., with TS ratio) severely violating the assumption of homoscedascticity, while following the subtraction method (right) dispersion is uncorrelated to fitted value satisfying the assumption of homoscedascticity.

1. These issues are a consequence of the non-linear properties of ratio conversions. The meaning of one unit in change in ratio is not constant across the range of values. This is particularly problematic for comparing changes above versus below 1.

Fig. 4.4: The correspondence between inhibition ration and inhibition by subtraction (left) as well as with TS MEPs (right) demonstrate concerning non-linearities induced by the ratio transform which may underlie the undesirable statistical properties identified above.
